# Comparison of Post-operative Functional Outcomes of Intertrochanteric Fractures Treated With Trochanteric Fixation Nail Versus Proximal Femoral Nail

**DOI:** 10.7759/cureus.86942

**Published:** 2025-06-28

**Authors:** Rabi R Prasad, Manu Gautam, Abhishek Sengupta, Hitesh Garg, Rahul Mali, Aruddha Sarkar, Joydeep Das

**Affiliations:** 1 Orthopaedics, Max Super Speciality Hospital, New Delhi, IND; 2 Orthopaedics, Max Super Specialty Hospital, New Delhi, IND; 3 Orthopaedics and Traumatology, Safdarjung Hospital, New Delhi, IND; 4 Orthopaedics, Vardhman Mahavir Medical College (VMMC) and Safdarjung Hospital, New Delhi, IND; 5 Orthopaedics and Traumatology, Max Super Specialty Hospital, New Delhi, IND; 6 Orthopaedics, Kalinga Hospital, Bhubaneswar, IND; 7 Orthopaedics and Traumatology, Vardhman Mahavir Medical College (VMMC) and Safdarjung Hospital, New Delhi, IND

**Keywords:** anterior thigh pain, geriatric hip fracture, harris hip scores, intertrochanteric fracture fixation, osteoporotic intertrochanteric fracture, pfn vs tfn

## Abstract

Background: Intertrochanteric femur fractures are common in the elderly and a growing public health concern. Surgical fixation remains the gold standard, with proximal femoral nail (PFN) and trochanteric fixation nail (TFN) being the most commonly used implants. This study compares the functional outcomes and complications of PFN and TFN in the Indian population.

Methods: A prospective study was conducted over 16 months at Max Super Specialty Hospital, New Delhi, involving 52 skeletally mature patients with intertrochanteric fractures. Patients were divided into two groups: PFN (n=26) and TFN (n=26). Functional outcomes were evaluated using the Harris Hip Score at six months. Pain was assessed using the visual analog scale (VAS) at six weeks and six months. Radiographs were used to assess fracture union and complications such as screw backout, varus collapse, and femoral canal impingement.

Results: No significant differences were found between the groups in terms of age, sex, operative time, or fracture type. The mean operating time was slightly shorter in the TFN group (42.4 min) than in the PFN group (45.7 min), though not statistically significant. Harris Hip Scores, VAS scores, and time to union were similar across groups (p > 0.05). Complication rates were low in both groups, with a slightly lower incidence of screw-related issues and femoral impingement in the TFN group.

Conclusion: Both TFN and PFN are effective and reliable options for intertrochanteric fracture fixation. While outcomes are largely comparable, TFN may offer marginal advantages in operative ease and implant-related complications.

## Introduction

Fractures of the hip are a significant public health issue and require extensive healthcare spending. It is estimated to increase from 26% of all hip fractures in 1990 to 37% in 2025, and to 45% in 2050 [1]. Intertrochanteric femur fractures account for 45% to 50% of fractures around the hip joint in elderly patients [2] with an incidence rate of 171 per 100000 [3].

Intertrochanteric fractures are defined as extracapsular fractures of the proximal femur that occur between the greater and lesser trochanter [4]. Improved life expectancy, particularly in emerging economies, is the primary cause of the increasing cases of hip fractures. The projections indicate that, by 2050, nearly half of all hip fractures will occur in Asia, mainly India and China. However, due to limited healthcare resources, almost two billion people globally do not have access to surgical care [5].

The best option for treating a hip fracture remains surgery since it can lead to anatomical reduction of the fracture, stable fixation, and maintenance of blood flow, allowing for early and full weight-bearing mobilization [6-11]. The quality of reduction is a crucial factor in the management of intertrochanteric fractures, regardless of the pattern of the fracture. In many cases, intra-medullary fixation is biomechanically superior to extra-medullary constructs. The restoration of cortical continuity is vital for the cortex to resist collapse in stable fractures [12,13]. Multiple implant options are available to the surgeon, out of which the trochanteric fixation nail (TFN) and the proximal femoral nail (PFN) are the two commonly used devices. TFN is a newer system with better design [14] and has been purported to have a better functional outcome.

Our study evaluates the functional outcome and complications of TFN and PFN for intertrochanteric fracture fixation in the Indian diaspora and has tried to identify any advantage one carries over the other.

## Materials and methods

After proper ethical clearance, a prospective study was conducted in the Department of Orthopaedic Surgery, Max Super Specialty Hospital, Shalimar Bagh, New Delhi, over a period of 16 months from January 2023 to June 2024. All patients who presented with intertrochanteric fractures were screened, and all those patients who were skeletally mature and were being operated within three weeks of injury were included in the study. Patients with non-united fractures, malunited fractures, pathological fractures and those without a six-month follow-up were excluded from the study. The patients were divided into two groups: one group underwent fixation using PFN and the second group was operated using the trochanteric fixation nail. Patients were recruited in each group in a sequential manner. Sample size calculation was done based on a study by Jha et al. [15], and a sample size of 52 patients was reached with a power of 0.14.

Patients’ demographic data was collected from the electronic medical system of the hospital, and it included the age, sex, injured side, duration of surgery, and AO classification of the fracture. Post-operatively, patients were assessed for functional outcome using the Harris Hip Score, which was evaluated at six months between the two groups. The visual analog scale (VAS) score was evaluated for anterior thigh pain post-operatively at six weeks and six months. Patients were also studied radiologically for time to union and complications were evaluated on radiograph at six months which included femoral canal impingement, coxa vara, proximal screw cut through, screw penetration, screw back out, and fracture non-union.

All the surgeries were performed by a single surgeon, under regional anesthesia, either spinal or epidural block unless contraindicated, on a fracture table in supine position under C-arm control. Patients were cleaned and draped, following which fracture reduction was assessed under C-arm and after achieving an acceptable reduction short cephalomedullary nail was inserted either PFN or TFN (AO) using the standard technique. All patients underwent distal locking using single screw in dynamic form and compression dressing was applied. Post-operatively, the patients were mobilized from POD1 partial weight bearing using support, which progressed to full weight bearing within one week. Knee and hip range of motion exercises were started from post-operative day 1.

**Figure 1:**
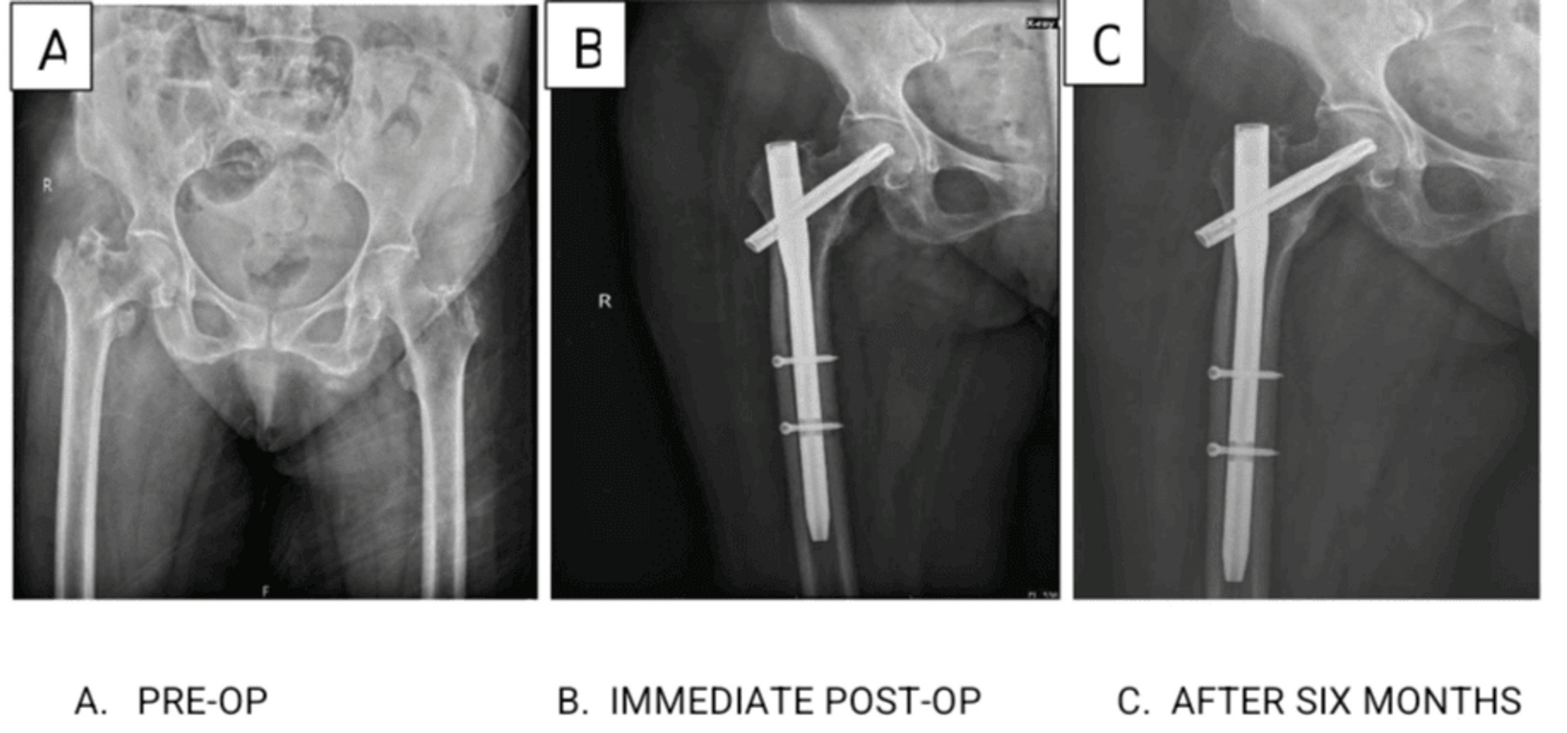
Post-operative X-ray of the patient operated using the proximal femoral nail.

**Figure 2:**
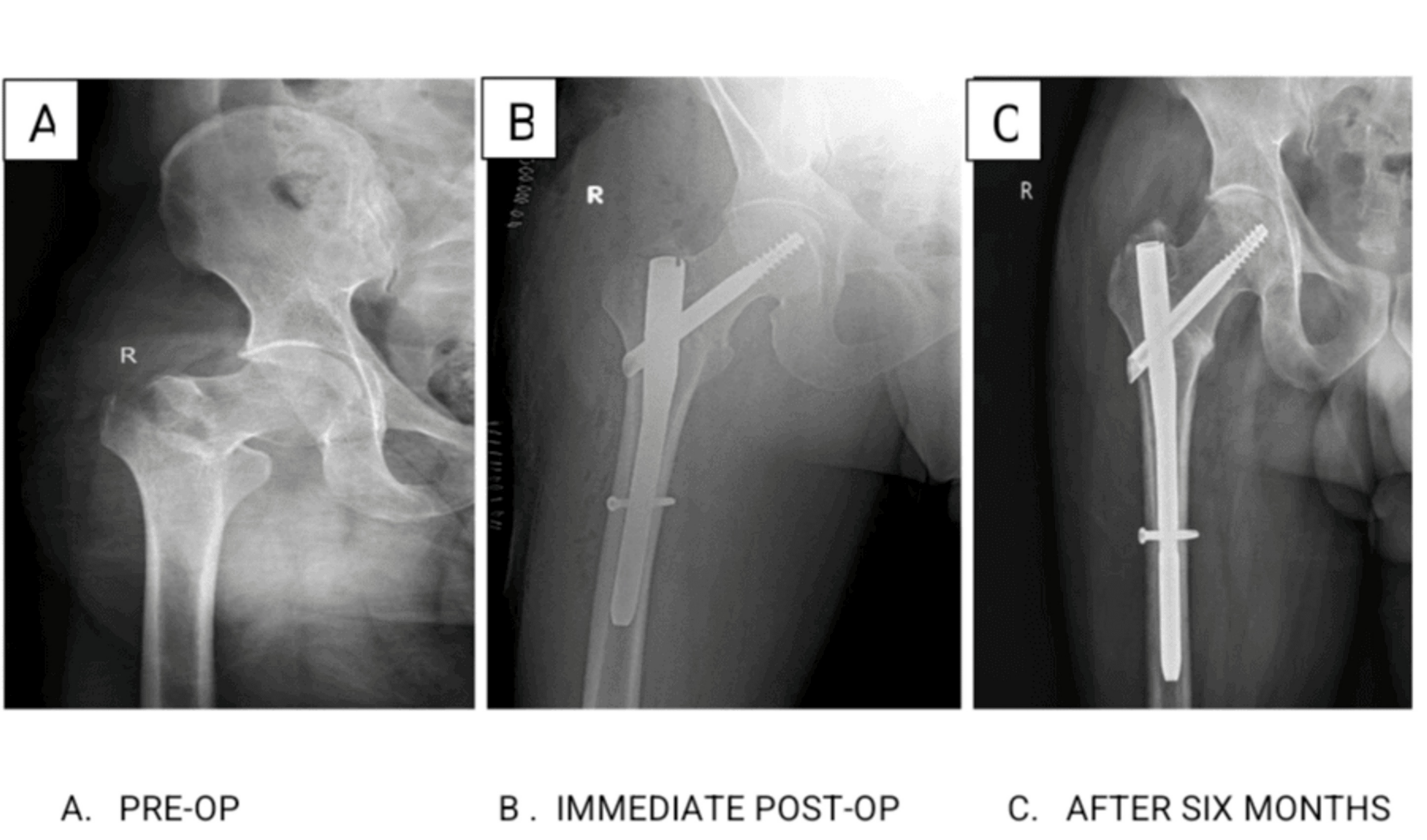
Post-operative X-ray of the patient operated using the trochanteric femoral nail.

Patients were followed up at two weeks, six weeks, three months, and six months and were evaluated for the VAS score and Harris Hip score at six months. Radiographic analysis was done regularly to assess for union and at the six-month point to assess for failure in the forms described previously (Figures 1, 2).

The Department of Biostatistics of Max Healthcare was consulted to provide support throughout the process. Data was analyzed using IBM SPSS Statistics for Windows, Version 27 (Released 2020; IBM Corp., Armonk, New York, United States). Descriptive data was reported for each variable. Summarized data was presented using tables and graphs. Data was normally distributed as tested using the Shapiro-Wilk W test (p-value was less than 0.05). The Mann-Whitney U test was used for comparison of VAS scores and hip scores. The chi-square test was used for categorical variables. A level of p<0.05 was considered statistically significant.

## Results

A total of 52 patients were included in the study with 26 participants in each group. There was no significant difference between age (p = 0.662) and sex distribution of the two groups. In the TFN group, 18 (69.2%) patients had fracture on the right side while in the PFN group, 15 (57.7%) patients had fracture on the right side. Both groups had no significant difference in the operating time (p = 0.204) with a mean operating time for the TFN group of 42.4±8.44 minutes, whereas the mean operating time for the PFN group is slightly longer at 45.7±9.82 minutes (Table 1).

**Table 1:** Demographic distribution of patients. TFN: Trochanteric fixation nail; PFN: proximal femoral nail

Variable	Category	TFN (n = 26)	PFN (n = 26)
Sex	Male (M)	12	14
	Female (F)	14	12
Mean Age		59.4 years	60.7 years
Preop Fracture Type	Type 1	6	1
	Type 2	10	8
	Type 3	10	17
Side of Injury	Right (R)	18	15
	Left (L)	8	11

The Harris Hip Score that was evaluated at six months had no significant difference between the two groups (p = 0.465). In addition, linear regression analysis of the components of the Harris Hip Score also showed no significant difference. The VAS score evaluated at six months for anterior thigh pain also demonstrated no significant difference (p= 0.421). The average time to union in the TFN group and the PFN group was 2.54±0.71 months and 2.71±1.01 months, respectively (p = 0.478) (Table 2).

**Table 2:** Six months post-operative Harris Hip Score of TFN and PFN groups. TFN: Trochanteric fixation nail; PFN: proximal femoral nail

Harris Hip Score	TFN	PFN
60-69	3	4
70-79	11	12
80-89	9	7
90-99	3	3
Total	26	26
Mean ±SD	78.7±7.47	77.1±8.34
		P= 0.465

The PFN group had one incidence of varus collapse, one incidence of screw penetration, and three incidences of screw back out, while the TFN group had one incidence of varus collapse and one incidence of screw back out; however, none of them reached a level of significance. There was only one case of femoral canal impingement in the TFN group while there were three cases in the PFN group; however, the difference was not significant. No other complications were observed between the two groups (Table 3).

**Table 3:** Complications observed in the TFN and PFN groups. TFN: Trochanteric fixation nail; PFN: proximal femoral nail

Complication	TFN (n = 26)	PFN (n = 26)
Varus Collapse	1	1
Cut Through	0	0
Screw Penetration	0	1
Screw Backout	1	3
Failure	0	0

## Discussion

Our study evaluated the difference in functional outcome between patients operated for intertrochanteric fractures using TFN and PFN. We also tried to assess the difference in anterior thigh pain between the two groups and the rate of complications. Our data demonstrated no significant difference in terms of functional outcome, anterior thigh pain, time to union, and post-operative complications between the two groups.

The majority of the patients had right-side involvement, with most patients classified as type 3 AO/OTA type fracture. This is in accordance with the findings of Kumar et al. [16]. This uniformity in fracture type distribution is crucial for comparing the outcomes, as it ensures that both groups had similar baseline fracture severity. The mean operating time was slightly longer for the PFN group (45.7 minutes) compared to the TFN group (42.4 minutes). This difference, although not statistically significant (p = 0.204), suggests that PFN might require slightly more operative time, potentially due to the technical nuances associated with its insertion. Studies by Morihara et al. [17] and Gadegone et al. [18] have similarly reported marginally longer operating times for PFN, attributing it to the additional steps involved in achieving optimal nail placement and securing distal locking screws [19].

The functional outcomes measured by the Harris Hip Score were slightly better in the TFN group compared to the PFN group, although this difference was not statistically significant (p = 0.465). This aligns with the findings of Gadegone et al. [18], who reported no significant difference in functional scores between the two groups. However, some studies, such as those by Huang et al. [19], have suggested that PFN may offer better functional outcomes in terms of earlier mobilization and reduced pain, although these differences were not observed in our study. There was no difference in the post-operative pain score of both groups; similar observations were drawn by Lenich et al. [20], who found no significant difference in pain levels between patients treated with PFN and those treated with TFN.

The incidence of varus collapse was identical in both groups, with only 3.8% of patients affected. Studies by Morihara et al. [17] and Huang et al. [19] have reported similar findings, suggesting that both fixation devices provide adequate stability with low rates of varus collapse and femoral complications. The incidence of screw penetration was higher in the PFN group (3.8%) compared to the TFN group (0%), although this difference was not statistically significant (p = 0.313). Screw backout was also more frequent in the PFN group (11.5%) compared to the TFN group (3.8%), but again, this difference was not statistically significant (p = 0.298). No failures were observed in either group, indicating that both devices provide robust fixation. These findings are consistent with those of Gadegone et al. [18] and Huang et al. [19], who reported low rates of mechanical complications with both TFN and PFN. Anterior impingement was more common in the PFN group compared to the TFN group, though not significantly. These findings were similar to those observed by Jha et al. [15]; they proposed that due to shorter height in the Indian population and an increased anterior bow of the femur, the PFN had an increased incidence of anterior impingement. The TFNA nail was introduced, as having different properties in material, length, variety, and design; the slightly better functional outcomes observed in the TFN group, although not statistically significant, might be attributed to the design features of the TFN, which include a more anatomical curvature and a smaller proximal diameter which may explain the lower rates of screw penetration and anterior impingement.

Our study is not without limitations. A short study duration with only six months of follow-up and a small sample size are the main limitations affecting our study. However, very few studies have compared the difference in functional outcome of PFN and TFN, and more robust research is required to achieve more conclusive evidence.

## Conclusions

In this comparative study of intertrochanteric fractures treated with TFN versus PFN, our findings reveal that both fixation methods yield similar and effective outcomes. The subtle edge observed in the functional outcomes and shorter operating times for the TFN group, although not statistically significant, suggests a potential preference for this device in certain clinical scenarios.

Notably, both TFN and PFN demonstrated robust performance in promoting fracture union, maintaining reduction, and minimizing post-operative pain. The incidence of complications such as screw penetration and backout was minimal, further highlighting the reliability of both devices. Given these results, the choice between TFN and PFN should be tailored to the specific fracture characteristics, patient profiles, and the surgeon’s expertise. Our study underscores the versatility and effectiveness of both devices, providing valuable insights for orthopedic surgeons in making informed decisions for the optimal management of intertrochanteric fractures. The nuanced advantages of TFN in operating time and functional outcomes offer a compelling case for its consideration, while PFN remains a strong and viable alternative, particularly in complex fracture patterns.
